# Adolescent Depressive Symptoms and Peer Dynamics: Distorted Perceptions in Liking and Disliking Networks

**DOI:** 10.3390/bs14111110

**Published:** 2024-11-19

**Authors:** Diego Palacios, Silvia Caldaroni, Christian Berger, Daniele Di Tata, Davide Barrera

**Affiliations:** 1 Society and Health Research Center, Facultad de Ciencias Sociales y Artes, Universidad Mayor, Santiago 7510041, Chile; 2 Millennium Nucleus for the Evaluation and Analysis of Drug Policies (nDP), Santiago 7560908, Chile; 3 Millennium Nucleus on Sociomedicine (SocioMed), Santiago 7560908, Chile; 4 Escuela de Educación, Facultad de Ciencias Sociales y Artes, Universidad Mayor, Santiago 7500994, Chile; 5 Department of Psychology, Sapienza University of Rome, 00185 Roma, Italy; silvia.caldaroni@uniroma1.it; 6 Escuela de Psicología, Pontificia Universidad Católica de Chile, Santiago 7560908, Chile; cberger@uc.cl; 7 Department of Developmental and Social Psychology, Sapienza University of Rome, 00185 Roma, Italy; daniele.ditata@uniroma1.it; 8 Department of Culture Politics and Society, University of Turin, 10153 Turin, Italy; davide.barrera@unito.it; 9 Collegio Carlo Alberto, 10124 Turin, Italy

**Keywords:** depressive symptoms, liking, disliking, RSiena, adolescence

## Abstract

Depression in adolescents has been linked to poor life outcomes, including suicidal ideation, peer victimization, and fewer friendships. Less is known about how depressed adolescents perceive their peer interactions. Based on the depression-distortion model, we expected that adolescents with depressive symptoms misperceive their social ties by being less likely to like some peers, and more likely to dislike other peers. An Italian dataset about adolescent relationships was used, including 275 first-year secondary school students (M age = 11.80, 46% female) in 12 classrooms across nine schools. Adolescents were asked to nominate classmates they liked and disliked. Longitudinal social network analyses (stochastic actor-oriented models) were conducted, including structural network effects (reciprocity, transitivity, indegree-popularity) and covariates such as gender, immigrant origin, and highest parents’ education level. The results indicated that adolescents with depressive symptoms were less likely to send liking nominations, and conversely, they were more likely to send disliking nominations than non-depressed classmates. Interestingly, adolescents with depressive symptoms were not more disliked or less liked by their peers. These findings seem to support the depression-distortion model by suggesting that, compared to non-depressed peers, adolescents with depressive symptoms misperceive their relationships by overstating negative relationships and underestimating positive ones.

## 1. Introduction

Depression is one of the most prevalent mental illnesses [1], and affects approximately 280 million people globally [2,3]. Adolescent depression is relatively high in mid-to-late adolescence [3], with prevalences ranging from 4%–5% [4] to 34% of adolescents reporting depressive symptoms [5]. In line with this, previous research in Italy has reported prevalence rates of depressive symptoms among adolescents ranging from 3% to 17% [6]. Specifically, it was estimated that 12% of Italian adolescents aged 11–13 reported experiencing moderate levels of depressive symptoms, while 6% and 3% exhibited moderately severe and severe levels of depression, respectively [6]. Moreover, a study involving a representative sample of Italian adolescents [7] found that the prevalence of internalizing symptoms increased with age, particularly among girls. Indeed, according to a recent study [6], 17% of Italian adolescents aged 14–17 were estimated to experience moderate depressive symptoms, while 9% and 8% suffered from moderately severe and severe depressive symptomatology, respectively.

Regarding socioeconomic status (SES), it is well documented that economic difficulties are associated with greater depressive symptoms, especially during adolescence [8]. This may be particularly true for immigrant youth, who often tend to live in more disadvantaged economic circumstances compared to their non-immigrant counterparts [9,10]. In this respect, previous studies conducted in European countries [11,12,13] have found a positive association between having a migrant background and the presence of internalizing symptomatology (i.e., depression and anxiety) during adolescence. Also, living in a rural setting in Italy may be another significant risk factor for the development of depressive symptoms [14]. From a developmental standpoint, rural and metropolitan environments offer distinct contexts that shape mental health outcomes differently [15]. Rural areas, often characterized by isolation and limited access to healthcare, increase psychological risks such as depression and anxiety [16,17]. A study by Gori and colleagues [14] found that Italian adolescents in rural settings exhibited a higher incidence of internalizing disorders, particularly somatic complaints, social, and cognitive problems, compared to their counterparts living in urban settings.

Depression is associated with deficits in several cognitive processes, such as emotional processing, attention, cognitive control, and memory [18]. Depressed individuals often exhibit cognitive distortions, which leads them to interpret neutral or ambiguous stimuli as threatening or hostile [19]. These distorted perceptions contribute to a negativity bias, where individuals are more likely to focus on negative aspects of their experiences while dismissing positive or neutral information. Adolescents with depressive symptoms are more likely to perceive ambiguous peer interactions negatively and interpret social cues as rejection, contributing to a cycle of poor peer relations [20].

During adolescence, social relationships become highly influential for social and mental development [21], identity formation, and (mal)adjustment [22,23]. In particular, peer relations play a crucial role in adolescents with depressive symptoms. Adolescents often define themselves and shape their identity based on peer interactions. They feel a growing need for closeness and become increasingly concerned about their status among peers [24,25]. Depressive symptoms can significantly impact peer relationships, with depressed adolescents often being less liked by their peers, though not necessarily less liking others themselves [26].

Being accepted or rejected by peers plays a crucial role in shaping young individuals’ behavior, and mental health in adolescence. While positive experiences can elicit feelings such as pride and respect and promote youth adjustment, negative experiences can have negative repercussions such as substance use [27] and internalizing symptoms [28]. Youth with depressive symptoms may experience difficulties asserting their needs with peers, consequently displaying less social initiative and becoming easily discouraged in challenging social situations [29].

In this developmental stage, the judgment of others is important in shaping adolescents’ feelings about themselves, forming a clear view of their personality, and navigating and establishing peer interactions [30,31]. Depressed individuals may believe that others see them as incompetent, undesirable, or unattractive, which might affect their relationships. The perception of rejection or acceptance by others, and not necessarily the actual rejection or acceptance, could affect their liking and disliking ties. For example, if depressed adolescents believe they are being treated poorly by peers, even without supporting evidence, their liking and disliking ties might be affected by this erroneous perception [32,33].

In this paper, we investigate the link between adolescents’ depressive symptoms and liking and disliking networks by examining whether students with depressive symptoms are more (un)likely to dislike and like other peers during a critical period of social development (e.g., the first year of secondary school). Unlike cross-sectional studies that provide a snapshot of associations at one point in time, our longitudinal network analysis tracks how liking and disliking ties evolve across multiple waves, offering insights into the formation of new ties and the maintenance of existing ones. Also, this network approach provides a comprehensive view of how individuals’ behaviors relate to their social connections, offering insights into the dynamics of relationships beyond an individual-level analysis [34].

### 1.1. Depressed Adolescents’ Perception of Themselves and Peer Status Relationships

Depressive symptoms may be rooted in a more extensive pattern of negative self-evaluation [35,36]. According to the *cognitive model of depression* [19,37], depressed individuals tend to adopt a negatively biased cognitive framework to organize and interpret their individual and social experiences. Consequently, this tendency leads to pervasive and distorted pessimistic beliefs about themselves, their relations with others, and the future.

Similarly, the *depression-distortion hypothesis* [38] suggests that depressed adolescents are more likely to assign a negative valence to social interactions than non-depressed adolescents and to perceive ambiguous peer experiences as stressful events [39,40]. For instance, depressed youth are more likely to perceive themselves as socially inept and unworthy of peer acceptance [41] and to believe that peers and friends are untrustworthy and hostile, tending to interpret social situations from a pessimistic perspective [42,43,44]. Specifically, adolescents with depression are more likely to attribute more negative and fewer positive meanings to ambiguous socio-emotional information [45,46,47], showing a reduced inclination to give a favorable interpretation of social cues [48]. They frequently question the authenticity of positive stimuli and disregard other favorable social cues, maintaining a pessimistic view of themselves and others’ intentions [42,43]. These negative biases and their reluctance to consider alternative interpretations could lead to anticipating adverse outcomes in future interactions, potentially contributing to social isolation [49].

Several studies have documented the distorted perception of the social context and deficits in social skills among depressed individuals. These misperceptions can compromise the quality of their peer relations [36,50]. Thus, symptoms commonly associated with depression, such as self-focused attention and negative cognition, significantly impact the expression of social behavior, potentially leading to a reduced likelihood of developing functional social networks [35].

Moreover, adolescents with depressive symptoms are more sensitive to interpersonal challenges, including conflict, criticism [39], and peer rejection [51]. In this regard, *rejection sensitivity* refers to a social–cognitive bias that reflects the tendency to perceive and expect rejection from others [52,53]. According to this model [54,55], these negative expectations are triggered in situations where the possibility of being rejected by another person exists. This leads individuals to interpret social cues as signs of rejection even when they may not indicate such, potentially eliciting a self-fulfilling prophecy that results in negative interpersonal outcomes [56]. Individuals with depression often show robust biases toward negative interpretations when processing ambiguous emotional stimuli [44], as well as exhibiting a reduced tendency to contradict negative interpretations [42,43].

Thus, despite others exhibiting good intentions and seeking positive social interactions with them, depressed adolescents may persist in maintaining initial negative impressions, rejecting alternative positive interpretations. Furthermore, there is evidence that depressed adolescents have a higher likelihood of befriending and liking other adolescents who are also depressed [57,58]. This is important because adolescence is a time of notable increase in intimacy and emotional disclosure within friendships, and adolescents often use peer interactions to appraise their self-worth. Thus, co-rumination and increased negative interpretation of life events may take place among youth with depressive symptoms, and this has been shown to lead to increased maladaptive self-blame for negative peer interactions [59]. Furthermore, depressed adolescents tend to gravitate towards others with similar symptoms, reinforcing negative cognitive patterns and potentially exacerbating their depressive symptoms [60]. This co-rumination can lead to maladaptive behaviors, further isolating them from wider peer networks.

### 1.2. Present Study

This paper adopts the depression-distortion model to investigate the association between depressive symptoms and liking and disliking networks in adolescence. Following evidence on depressed adolescents’ interpretation of social interactions as distressing and negative in valence [39], we propose that adolescents exhibiting higher depressive symptoms will create/maintain fewer liking ties to peers (Hypothesis 1). We also expect they will create/maintain liking ties, especially with peers with similar depressive symptoms (Hypothesis 2). Furthermore, as the rejection-sensitivity model suggests that students with depressive symptoms are more likely to anticipate rejection by their peers, we hypothesize that they will create/maintain disliking ties with a higher number of classmates than peers without depressive symptoms (Hypothesis 3). We also controlled for adolescents’ similarity in immigrant origin and parent educational levels, as there is evidence that both can positively relate to positive relationships such as friendships and liking [61,62].

## 2. Materials and Methods

### 2.1. Sample

The data come from 501 first-year secondary school students (M age = 11.58, SD age = 0.85, 46% female) in 23 classrooms across nine schools [63]. The average class size was 22 students (ranging from 17 to 25 students). The schools were located throughout the urban area of Turin, with an even distribution across different neighborhoods. Approximately 54% of the students were of Italian origin, 9% had one parent of foreign origin, and 35% had both parents of foreign origin. According to the Ministry of Education, about 30% of students in this age group in Turin hold foreign nationality [64]. Data were collected in three waves: the first in December 2015, the second in March 2016, and the third in June 2016.

These data were collected by a private foundation, Fondazione Scuola of Compagnia di San Paolo, and it was part of a combined effort between a local project (“*Provaci ancora Sam*”, SAM hereafter) and the European project 2young2fail [Erasmus project 2014-1-IT02-KA201-003609], which aims to decrease school dropout in secondary schools. The region’s minister of education endorsed this project. In Italy, students enroll in secondary school when they are approximately 11 years old. The classroom composition is constant throughout the year and does not change by subject. Thus, students had (almost) all subjects with the same classmates. The anti-dropout program (SAM) was active in about half of the classrooms and involved extracurricular activities to foster student engagement. In total, 23 classes were included in the data collection: 10 SAM classes and 13 non-SAM classes.

We analyzed three waves of data, with three months between assessments. This interval adequately assesses potential peer network changes [62,65]. Furthermore, as data collection began two months after the start of the first year of secondary school, we captured the social network at its origin when many new relations were developing and, therefore, changes between waves were even more likely. The final sample of twelve classrooms (277 students) was selected based on the proportion of missing data for each classroom (10% on average in the three waves; range: 4–18%). We excluded ten classrooms due to their high levels of missing data (higher than 20% on average in the three waves; range: 23–42%, see details about covariates at the classroom level in Table S1 in the Supplementary Materials); this was problematic for the type of social network analysis we employ (i.e., stochastic actor-oriented models (SAOM, [66]; please see the “Analytical Strategy” section below), as simulations may become unstable, and estimated parameters may not be substantively reliable anymore [65]. The included (*M* = 2.41, *SD* = 0.69) and excluded classrooms (*M* = 2.48, *SD* = 0.70) did not differ regarding depressive symptoms (*t*(343.88) = −1.01, *p* = 0.30) or gender distribution (*M_boys included_* = 0.53, *SD_boys included_* = 0.50; *M_boys excluded_* = 0.50, *SD_boys excluded_* = 0.50; *t*(282.21) = 0.55, *p* = 0.58). There were statistical differences regarding immigrant origin (*M_included_* = 0.37, *SD_included_* = 0.48; *M_excluded_* = 0.59, *SD_excluded_* = 0.49; *t*(279.29) = −4.34, *p* < 0.001) and parents’ educational levels (*M_included_* = 3.94, *SD_included_* = 1.28; *M_excluded_* = 3.62, *SD_excluded_* = 1.20; *t*(280.8) = 2.30, *p* = 0.02), where included classrooms exhibited a lower percentage of students of an immigrant origin and parents with higher educational levels on average, compared to excluded classrooms. However, the results obtained from the excluded classroom were not substantively different from the included ones (see Table S3 in the Supplementary Materials).

### 2.2. Procedure

The questionnaires were administered using custom-made software running on tablets, monitored by a teacher and two research assistants in the classroom during data collection. At the start of the study, the software imported each classroom register list so that each participant could select network ties by simply clicking on names in the list. At the end of the questionnaire, upon clicking “send,” the tablet visibly deleted all names from the data to show students that the study was truly anonymous.

The project was endorsed by the regional office of the Minister of Education, and passive parental consent was sought before data collection. No parents raised objections to children’s participation, likely due to the support from the regional minister. Participation in the study was voluntary, and students could withdraw anytime. They were assured that the questionnaire was anonymous and that their responses would be confidential.

### 2.3. Variables

*Liking and Disliking networks* (waves 1, 2, and 3). Participants were asked to nominate an unlimited number of classmates from a drop-down menu with a complete non-alphabetical class list that fitted with these two descriptors (peer nominations): “Which of your classmates do you find likable?” and “Which of your classmates do you find dislikable?”, respectively. Adjacency matrices were created for each classroom on each assessment representing each type of network, where nominations were coded 1, and non-nominations were coded 0. The average number of nominations for liking was around 11 in the first wave, 11.06 in the second wave, and 10.92 in the third wave. The average number of nominations for disliking was around 2.59 in the first, 3 in the second, and 3.18 in the third wave.

*Depressive symptoms* (wave 2). Students answered a 6-item scale on the frequency of depressive symptoms (from 1 = never to 5 = always) adapted from Kandel and Davies [67]. The items address how often students “feel too tired to do anything”, “feel sad”, “have difficulty falling asleep”, “have little hope in the future”, “feel tense and nervous”, and “worry too much about everything” (*M*t2 = 2.40, *SD*t2 = 0.68). The scale was found to be reliable (6 items; α = 0.70). We also conducted a Confirmatory Factor Analysis (CFA) to check the fit of a one-dimensional structure, resulting in a good fit of the items (CFI = 0.98; RMSEA = 0.049; SRMR = 0.03; x2(9) = 17.52; *p* < 0.05).

*Gender*. Students were asked about their gender, coded 0 for girls (54%) and 1 for boys (46%).

*Immigrant origin.* Students were asked about the country in which their parents were born. We recorded the original information distinguishing two groups based on whether at least one parent was born abroad [68]: students without (63%) and with immigrant origin (37%).

*Parents’ education level*. Students were asked about their parents’ education level. We took the highest education level of any of the parents. The categories were (1) no formal qualification, (2) elementary, (3) lower secondary school, (4) upper secondary school, and (5) university degree (*M*t1 = 3.92, *SD*t1 = 1.28).

### 2.4. Analytical Strategy

Studying adolescent peer relations in schools requires methodological approaches beyond the exclusive analysis of the individual by incorporating the relationships (ties) between actors within the classroom. Social network research emphasizes that the position of an actor (e.g., student) in a network determines, in part, the opportunities and constraints they encounter and thus plays a vital role in their behavioral outcomes. This study used stochastic actor-oriented models [66]. Likely trajectories between observations were modeled using data from the first wave as the initial reference point. The model estimates were derived through an iterative simulation process based on a Markov Chain approach, which reflects the magnitude of the effects included in the model. The unstandardized estimates, similar to regression coefficients in logistic regression, indicate the strength of each effect in the formation or persistence of a tie. The model parameters were evaluated using t-ratios, calculated as the parameter estimate divided by its standard error. Specifically, we used the stochastic actor-oriented models for analyzing the effects of depressive symptoms on liking and disliking networks while controlling for network structural effects (density, reciprocity, and transitivity) and relevant covariates (gender, immigrant origin, and highest parents’ education level).

To address missing data due to non-response, we utilized RSiena’s default missing-data handling method. Participants who joined or left the classroom network between time points were managed using structural zeros, representing impossible nominations. The model estimation was conducted for each classroom individually, employing the Method of Moments estimator with 5000 iterations in phase 3 to compute standard errors. The estimation process was divided into two steps. First, each classroom was analyzed separately, ensuring the algorithm achieved good convergence. The convergence criteria for the analyses included an overall maximum convergence ratio below 0.25 and individual t-ratios converging to less than 0.10 in absolute value [65]. In the second step, results from all classrooms were combined through a meta-analysis using the Snijders–Baerveldt test [69]. This meta-analysis tested the mean and variance of parameter estimates across classrooms, allowing for population-level inferences.

### 2.5. Model Specification

*Structural network effects*. These effects were included to capture the basic tendencies of actors to form and maintain liking and disliking networks. Density describes the tendency of actors to establish liking and disliking relationships. Reciprocity is the tendency to reciprocate liking and disliking relationships (referring to forming mutual ties). For liking networks only, we included the tendency to like classmates who are liked by peers that I like (transitivity). Finally, we had the indegree-popularity effect, representing the tendency of actors who receive many nominations to receive more nominations over time. All those effects were included to control network mechanisms common in adolescent school networks [70].

*Covariates*. The alter, ego, and similarity effects for depressive symptoms were included. Ego effects refer to the number of nominations an individual sends, alter effects concern the number of nominations an individual receives, and similarity effects refer to how similar the two actors (ego and alter) are with respect to a given variable (i.e., how liked or disliked they are). These effects capture the impact of covariates on received (more or less) liking and disliking nominations (alter or “popularity” effect), given liking and disliking nominations (ego or “activity” effect), and having liking and disliking relationships based on covariate similarity, respectively. Also, sex, immigrant origin, and parents’ education level were included as control variables by including the same sex and immigrant origin effects and the similarity effect for parents’ education level.

In the liking network analysis, the “depressive symptoms ego” effect was used to test Hypothesis 1; the “depressive symptoms similarity” effect was used to test Hypothesis 2, and in the disliking network analysis, the “depressive symptoms ego” effect was used to test Hypothesis 3.

## 3. Results

### 3.1. Descriptive Analyses

#### 3.1.1. Descriptive Analyses for Depressive Symptoms

We compared depressive symptoms by gender and immigrant origin. There was no significant difference in depressive symptoms between boys (*M* = 2.51, *SD* = 0.73) and girls (*M* = 2.36, *SD* = 0.65) (*t*(208.03) = 1.60, *p* = 0.110). We also compared depressive symptoms between students without (*M* = 2.40, *SD* = 0.70) and with immigrant origin (*M* = 2.48, *SD* = 0.68), finding no statistically significant difference (*t*(179.66) = −0.86, *p* = 0.393) in depressive symptoms between these two groups. Finally, we compared depressive symptoms among different parents’ education levels, finding no statistically significant difference (F(4, 306) = 1.61, *p* = 0.172) in depressive symptoms based on the parents’ educational level.

#### 3.1.2. Descriptive Analyses for Liking and Disliking Networks

Table 1 presents information on liking and disliking networks by immigrant origin and gender. Overall, the average sent (outdegree) and receiving (indegree) liking and disliking nominations were similar in roughly all three waves. We ran multiple *t*-tests to compare this. There were no significant differences in the outdegree for the liking network between students without and with an immigrant background in wave 1 (*t*(168.38) = 1.09, *p* = 0.278), in wave 2 (*t*(181.61) = −0.64, *p* = 0.521), and in wave 3 (*t*(173.65) = −0.08, *p* = 0.935). For the indegree in the liking network, we did not find differences in wave 2 (*t*(191.01) = 0.87, *p* = 0.386) and wave 3 (*t*(186.75) = 0.94, *p* = 0.348), but in wave 1, students without an immigrant origin (*t*(184.31) = 2.11, *p* = 0.036) had significantly higher indegree scores than students without an immigrant background. Regarding disliking networks, there were no significant differences in outdegree between students without and with an immigrant background in wave 1 (*t*(182.4) = 0.73, *p* = 0.464), in wave 2 (*t*(182.4) = 0.73, *p* = 0.464), and in wave 3 (*t*(197.96) = 1.25, *p* = 0.213). For the indegree in the disliking network, we did not find differences in wave 1 (*t*(184.58) = −1.62, *p* = 0.107), and wave 3 (*t*(178.5) = −1.18, *p* = 0.238), but in wave 2, students with an immigrant origin (*t*(184.58) = −1.62, *p* = 0.107,) had significantly higher indegree scores than participants without immigrant origin.

There were no significant differences in outdegree for the liking network between boys and girls; not in wave 2 (*t*(238) = 0.07, *p* = 0.946) and 3 (*t*(236.16) = 0.87, *p* = 0.386), but in wave 1 (*t*(237.13) = 2.03, *p* = 0.044), girls had significantly higher outdegree scores than boys. No differences were found for indegree in waves 1 (*t*(237.9) = −0.12, *p* = 0.908), 2 (*t*(237.9) = −0.12, *p* = 0.908) and 3 (*t*(229.06) = −1.14, *p* = 0.254). Regarding disliking networks, there were no differences in outdegree between boys and girls in wave 1 (*t*(213.37) = −0.76, *p* = 0.447), wave 2 (*t*(233.77) = 0.34, *p* = 0.731), and in wave 3 (*t*(226.08) = 1.49, *p* = 0.138). The differences appeared when comparing indegree in disliking networks with boys being consistently more disliked than girls in wave 1 (*t*(237.85) = −2.24, *p* = 0.026), 2 (*t*(237.54) = −2.46, *p* = 0.015), and 3 (*t*(234.94) = −2.81, *p* = 0.005).

Table S2 in the Supplementary Materials describes the changes in the liking and disliking ties between the three waves. The Jaccard index indicated how stable these relationships were between each period (e.g., period 1 comprises the change between waves 1 and 2). For the liking networks, stability was moderate, with about 51% of liking ties staying the same between the first and second wave and 57% staying the same between the second and third wave. For the disliking networks, stability was lower, with only 25% and 32% of the dislikes remaining the same between the two periods. Regarding the patterns of change in liking networks, most liking ties were maintained rather than dissolved (ended) or created (new liking). For disliking networks, the changes were more balanced, with similar relationships being maintained, dissolved, or newly created over time. Jaccard values less than 0.2 (less than 20% of tie stability) can lead to doubts about the assumption that the network change process is gradual [62].

These findings suggest that while gender, immigrant origin, and parental education did not significantly influence depressive symptoms, there are notable differences in liking and disliking networks. First, boys appeared to experience higher rejection levels, as they were more disliked across all waves. Second, students with an immigrant background may have faced social integration challenges, as reflected by the lower liking nominations in wave 1 and higher disliking nominations in wave 2.

### 3.2. Longitudinal Network Analyses

Table 2 shows the meta-analysis results for twelve classrooms (275 students). The aggregated results indicate that liking networks were less likely to be reciprocated (est. = −0.20, *p* < 0.001), but, interestingly, they exhibited a transitive structure (est. = 0.12, *p* < 0.001), indicating that if I like a classmate and he/she likes a third one, I will tend to like that third peer. The significant negative effect of indegree-popularity (est. = −0.03, *p* = 0.02) indicates that well-liked peers were less likely to receive additional liking nominations over time. Regarding the covariates, adolescents liked peers of the same gender (est. = 0.36, *p* < 0.001) and of a different immigrant origin (est. = −0.18, *p* < 0.001). For depressive symptoms, adolescents liked peers with similar levels of depressive symptoms (est. = 0.21, *p* = 0.04); this provides empirical support for Hypothesis 2, and students with higher levels of depressive symptoms were less likely to like other classmates (est. = −0.06, *p* < 0.001), providing support for Hypothesis 1. No effects of depressive symptoms on receiving nominations were found (est. = −0.03, *p* = 0.48).

Regarding the disliking networks, these relationships were reciprocal (i.e., I dislike others who dislike me; est. = 0.32; *p* < 0.001), and rejected students increased their dislike nominations over time (positive indegree-popularity, est. = 0.13, *p* < 0.001). Adolescents disliked peers of a different gender (est. = −0.26, *p* = 0.02) and of the same immigrant origin (est. = 0.13, *p* = 0.04). Finally, we found that students with depressive symptoms were more likely to dislike other classmates (est. = 0.29, *p* = 0.01), providing support for Hypothesis 3. No effects were found for alter (est. = −0.04, *p* = 0.20) and similarity effects (est. = −0.02, *p* = 0.91), indicating that adolescents with high depressive symptoms were not more rejected than other peers without depressive symptoms and that similar depressive symptoms were not a significant factor for rejecting peers.

**Figure 1:**
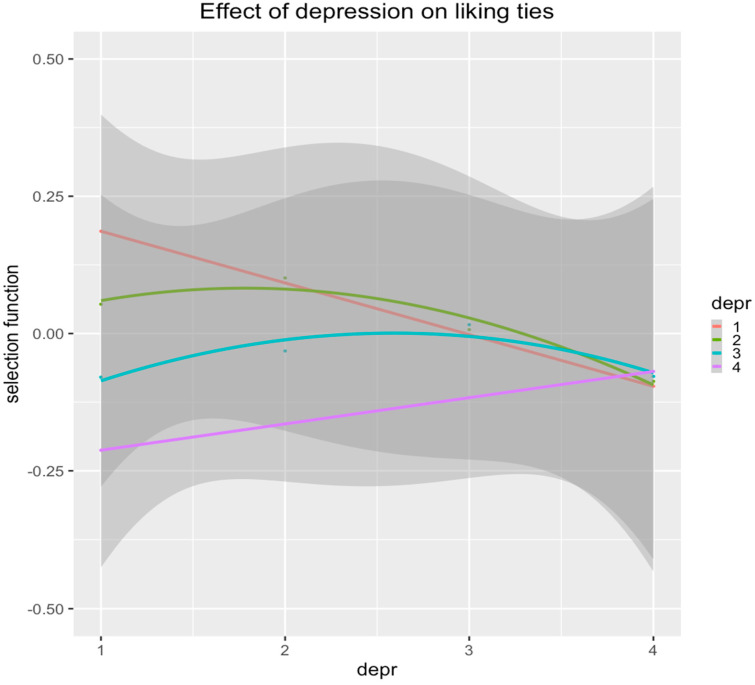
Liking-ties selection of adolescents’ (ego) on peers (alter) based on levels of depressive symptoms. *Note*. The selection function indicates the preference for selecting liked peers combining ego and alter depressive-symptom levels (x-axis, and curve colors, respectively). The values of ego and alter represent null (1), low (2), moderate (3), and high (4) values of depressive symptoms.

To go deeper into the results of Hypotheses 1 and 2, we constructed a selection plot for liking networks (Figure 1), which shows an average classroom in the sample (closer to meta-analysis results for ego, alter, and similarity depressive symptom effects). This figure represents the preference of liking networks (y-axis, selection function) depending on the depressive symptoms’ levels of both ego (color curves) and alter (x-axis) depressive symptom levels.

The salmon-colored line represents adolescents (egos) who scored lowest on the depression scale (i.e., null); their preference was highest (value = 0.19) for peers (alters) who also scored lowest on the depression scale and was lowest (value = 0.19) for peers (alters) who scored highest on the depression scale. Overall, this figure suggests that students tend to prefer liking peers with similar levels of depressive symptoms (as the maximum values of preference of each curve (color) are at the same values of depressive symptoms), particularly for adolescents with lower levels of depressive symptoms (salmon- and green-colored lines). Also, students with higher levels of depressive symptoms (turquoise- and purple-colored lines) were less attracted to liking other peers (negative values of the selection function).

### 3.3. Sensitivity Analysis

We ran the exact model specification for each classroom to check whether the results differed when analyzing the excluded classrooms. Then, we combined the results using the same meta-analytical procedure. Only one classroom was removed, due to its lack of convergence. The model results (see Table S3 in the Supplementary Materials) were very similar for the three effects related to our hypotheses.

## 4. Discussion

This study contributes to investigating the link between youth depressive symptoms and peer relations, in particular, liking and disliking networks. Its findings indicated that adolescents with depressive symptoms, compared to non-depressed peers, sent a higher number of disliking nominations and a lower number of liking nominations. Moreover, adolescents were more likely to like peers with similar levels of depressive symptoms, but this effect was mainly present among adolescents with lower or null levels of depressive symptoms. Furthermore, compared to peers with no depressive symptoms, we found that adolescents with depressive symptoms did not receive either a lower number of liking nominations or a higher number of disliking nominations.

Central to this paper was the depression-distortion model, which posits that individuals with depression may have a distorted view of their social interactions, often perceiving them more negatively than they objectively are [71]. This theoretical framework provides a critical lens to understand our findings, particularly the relationship between depressive symptoms and perceptions of disliking within peer networks. Our results align with this model, suggesting that adolescents with higher levels of depressive symptoms may perceive themselves as being disliked by their peers, even when such perceptions may not be entirely accurate. This distortion highlights the importance of addressing depressive cognitions when evaluating social networks in adolescence.

Together, these results suggest a potential discrepancy between their actual and perceived social status [19]. This incongruency may derive from negative interpretations of their peer relations and higher social-rejection expectations [72]. Adolescents with depressive symptoms might misperceive their social interactions by focusing on their negative aspects, consequently making decisions consistent with this view. In this line, Humenny et al. [73] argue that adolescents’ loneliness is shaped more by their meta-perceptions—how they believe they are viewed by others—than by their actual social status. Adolescents with depressive symptoms often have distorted self-perceptions, believing they are rejected by peers even when this is not the case, which intensifies their loneliness. Similarly, Bleckmann et al. [74] found that meta-perception of liking increases over time, but those initial perceptions are strongly influenced by neuroticism and self-esteem. Adolescents with lower self-esteem or higher neuroticism tend to expect rejection, exacerbating their depressive symptoms and further distorting peer relationships, reinforcing a cycle of social withdrawal and negative self-evaluation.

These adolescents may also have a distorted view of themselves as unworthy of acceptance or they may lack social self-efficacy to interact confidently with peers [36]. In this line, previous studies showed that children who experience depression often tend to view their status more negatively compared to their peers, feel less accepted by their peers, and report lower friendship quality with their best friends when compared to non-depressed children [75,76,77]. Moreover, a meta-analysis on friendships and loneliness and depressive symptoms in childhood and adolescence [78] found that loneliness is more directly tied to the number of friendships, whereas depressive symptoms are more closely linked to the quality of friendships. The findings of the present study are aligned with these studies, suggesting that adolescents with depressive symptoms may perceive the quality of their relationships more negatively, even if they are not socially isolated or lacking in friends.

Our findings are also consistent with studies conducted among college students [79], indicating that individuals with depressive symptoms and reduced self-esteem tend to perceive that others like them less and are less interested in maintaining future contact than is indeed the case. Moreover, those same students exhibited a lower tendency to assume that partners return one’s feelings of liking and acceptance (reciprocity bias) [79].

Simultaneously, depressed youth could overestimate their contribution to negative interactions [41,80] and to search for information, thus confirming their negative self-views [71]. Moreover, this pessimistic view may also affect the perception of others as being hostile or untrustworthy [41]. As suggested by the Scar hypothesis [51], they may be more sensitive to the perception and recall of social rejection due to biased cognitive functioning. As a result, these erroneous perceptions may lead to expectations of rejection [72] and to consequent behavioral responses such as proactively rejecting others in anticipation of one’s own rejection, as shown by the tendency of students with higher depressive symptoms to send more disliking than liking nominations.

These preferences may underlie an inclination towards adolescents with the same perceptions, attitudes, or behaviors, as this minimizes uncertainty and promotes positive reinforcement during social interactions [81,82]. This inclination aligns with previous findings showing that depressive symptoms can spread through peer influence and contagion, especially among adolescents. For example, van Zalk et al. [83] found that peers influence each other’s depressive symptoms through mechanisms like failure anticipation [84].

At the same time, these preferences may be a risk factor for adolescents experiencing depressive symptoms, since they may seek social support and reassurance [82,84] from other non-depressed peers, but they end up having relationships with other similarly depressed peers, thereby reinforcing their negative attributions (e.g., self-blaming) or engaging in problem-focused conversations and co-rumination [59]. In this line, a recent study by Qin et al. [85] emphasizes the dual role of vulnerable friendships, where depressed adolescents tend to seek out similarly vulnerable peers, which may reinforce negative perceptions and behaviors.

Unlike studies relying on self-reported data alone [20,60,86], this social network analysis provides a broader view of peer interactions. While some studies (e.g., [20,60]) trace the longitudinal relationships between peer rejection and depressive symptoms, our social network analysis focuses on peer dynamics within the classroom setting, and consequently controls for common network mechanisms for liking and disliking networks such as reciprocity (e.g., I like peers who like me), transitivity (e.g., I like classmates who are liked by peers that I like), or self-reinforcing mechanisms (e.g., liked peers tend to increase their received liking ties over time). This approach offers insights into the evolution of liking and disliking networks. These differences in methodology may explain variations in findings across studies, such as previous findings that show that depressed adolescents are less liked by their peers, though not necessarily they like other peers less [26].

We found unexpected results for immigrant origin, where adolescents liked peers of a different immigrant origin and disliked peers of the same immigrant origin (students with and without an immigrant origin). These results might have occurred because the group of students with an immigrant origin is generally quite heterogeneous within each classroom. In most classrooms, there is no homogeneous (i.e., from the same foreign country) group of students with an immigrant origin large enough to form a clique. Furthermore, within this group, the most common nationalities are Morocco, Romania, Albania, China, Egypt, and the Philippines. While it is tempting to think that students with an immigrant origin tend to cluster together, those groups are culturally different, and it is possible that, instead of this, they do not like each other very much. Therefore, future studies should have enough subgroups from different nationalities of a reasonable size to investigate this further, as adolescents from the same nationality or ethnic origin tend to cluster together (e.g., [87]).

It is important to acknowledge the limitations of this study. First, we focused on the impact of depressive symptoms on peer relations. However, a more comprehensive understanding could be achieved following a transactional approach [88] by assessing longitudinally the interplay between depressive symptoms and peer relations over time (see, for example, Kiuru et al. [89]). Peer rejection is not only a consequence of depressive symptoms, but can also exacerbate them over time, creating a feedback loop where depression leads to further social withdrawal and peer rejection [86]. Unfortunately, this study only contained one measurement of depressive symptoms. Second, the reliance on a self-report measure of depressive symptoms could introduce the potential for inaccuracies and desirability bias in respondents’ answers. Future research should, therefore, analyze depressive symptoms using peer-perceived measures or clinical assessments and mixed methodologies, to provide a more comprehensive view. Third, since all participants are adolescents from a specific region in Italy with unique cultural and social characteristics, generalizing these results should be done with caution. Turin, the fourth largest city in Italy, provides an urban context, which may limit the applicability of these findings to less-urban areas where the prevalence of depression is potentially higher [14]. However, in a sense, this represents a conservative sample, as the effects observed in an urban setting could be amplified in rural areas, where depression is more prevalent. Fourth, the duration and frequency of the assessments regarding the variables (e.g., depressive symptoms and peer relationships) depends on the expected change in those variables over time. Then, for instance, it would be reasonable to have a shorter interval for liking and disliking relationships than for depressive symptoms. Recent longitudinal studies of adolescents’ networks on depressive symptoms and friendships have been conducted using between half-year and yearly intervals and between three and five measurement points [83,85,90]. Future studies should evaluate the appropriate time and frequency for measuring those variables. Finally, as gender plays a significant role in moderating this relationship, with boys’ depressive symptoms being more closely linked to achievement-related status and girls’ symptoms being associated with affection-related status [57,91], future research should incorporate a gender-focused analysis to better understand how different dimensions of peer status interact with depressive symptoms in adolescent populations.

## 5. Conclusions

This paper sheds light on the relationship between adolescents who experience depressive symptoms and their social ties by investigating their liking and disliking networks and examining whether students with depressive symptoms are more (un)likely to dislike and like other peers. The study revealed that adolescents experiencing depressive symptoms sent more disliking nominations and fewer liking nominations compared to their non-depressed peers. Additionally, adolescents tended to prefer peers with similar levels of depressive symptoms, although this was primarily observed in adolescents with little-to-no depressive symptomatology. Interestingly, unlike their peers without depressive symptoms, adolescents with depressive symptoms did not receive fewer liking nominations or more disliking nominations [92].

Adolescents with depressive symptoms are highly vulnerable due to their mental health condition, which can distort their social interactions. According to the depression-distortion model, they are more likely to misinterpret social cues, perceiving peers as less accepting or even hostile, which can lead to expectations of rejection. This anticipation of rejection further damages their self-perception and peer relationships, often leading to social withdrawal or negative behaviors that may result in actual rejection [51,52].

This lack of recognition limits the support they receive from teachers and school staff, which is crucial for their mental health and peer relationships [58,59]. Addressing both the psychological and social needs of adolescents experiencing depressive symptoms should be a priority for educators and mental health professionals, to ensure better outcomes for this at-risk group.

## Figures and Tables

**Table 1:** Liking and disliking network descriptives by immigrant origin and gender.

Variable	Mean w1	N w1	SD w1	Min w1	Max w1	Mean w2	SD w2	Min w2	Max w2	Mean w3	SD w3	Min w3	Max w3
Liking sent nominations (outdegree) by immigrant origin													
No Immigrant origin	13.01	151	6.11	0	24	11.36	6.99	0	24	12.01	6.81	0	23
Immigrant origin	12.06	89	6.84	0	23	11.97	7.13	0	24	11.23	7.13	0	24
Liking received nominations (indegree) by immigrant origin													
No Immigrant origin	11.66	151	3.49	1	19	11.65	3.75	0	20	3.70	3.61	0	14
Immigrant origin	10.67	89	3.50	1	19	11.22	3.60	2	19	3.03	3.27	0	20
Disliking sent nominations (outdegree) by immigrant origin													
No Immigrant origin	3.19	151	4.24	0	24	3.27	3.65	0	19	10.97	3.31	1	18
Immigrant origin	2.66	89	3.66	0	22	2.91	3.70	0	24	11.56	4.63	0	21
Disliking received nominations (indegree) by immigrant origin													
No Immigrant origin	2.36	151	2.34	0	12	2.62	2.68	0	15	2.52	3.08	0	15
Immigrant origin	2.87	89	2.35	0	13	3.42	3.15	0	15	3.65	3.14	0	16
Liking sent nominations (outdegree) by gender													
Girls	13.54	112	5.67	0	24	11.62	6.55	0	24	12.01	6.81	0	23
Boys	11.89	128	6.90	0	24	11.55	7.46	0	24	11.23	7.13	0	24
Liking received nominations (indegree) by gender													
Girls	11.27	112	3.23	1	18	11.18	3.10	2	19	10.97	3.31	1	18
Boys	11.32	128	3.77	1	19	11.77	4.13	0	20	11.56	4.63	0	21
Disliking sent nominations (outdegree) by gender													
Girls	2.79	112	2.92	0	13	3.22	3.12	0	12	3.70	3.61	0	14
Boys	3.17	128	4.81	0	24	3.06	4.09	0	24	3.03	3.27	0	20
Disliking received nominations (indegree) by gender													
Girls	2.19	112	2.20	0	11	2.44	2.57	0	15	2.52	3.08	0	15
Boys	2.86	128	2.45	0	13	3.34	3.08	0	15	3.65	3.14	0	16

**Table 2:** SAOM results for liking and disliking networks (12 classrooms).

Effect	Est.	SE	*p*	Sigma	Q	Qp
*Liking networks*						
Rate period 1	19.62	2.06	<0.001	4.10	18.79	0.07
Rate period 2	12.20	0.83	<0.001	1.64	21.84	0.03
outdegree	−1.14	0.07	<0.001	0.21	43.73	<0.001
reciprocity	−0.20	0.06	<0.001	0.15	21.79	0.03
transitive triplets	0.12	0.01	<0.001	0.03	68.42	<0.001
indegree-popularity	−0.03	0.01	0.02	0.04	54.51	<0.001
same sex	0.36	0.04	<0.001	0.11	11.37	0.41
same immigrant origin	−0.18	0.05	<0.001	0.12	22.08	0.02
parents’ education similarity	0.09	0.10	0.36	0.24	23.61	0.01
depressive symptoms alter	−0.03	0.04	0.48	0.09	24.96	0.01
depressive symptoms ego (Hypothesis 1)	−0.06	0.02	<0.001	0.06	5.91	0.93
depressive symptoms similarity (Hypothesis 2)	0.21	0.10	0.04	0.23	19.14	0.06
*Disliking networks*						
Rate period 1	7.01	0.93	<0.001	2.42	37.38	<0.001
Rate period 2	6.72	0.75	<0.001	0.76	46.73	<0.001
outdegree	−1.28	0.08	<0.001	0.22	43.96	<0.001
reciprocity	0.32	0.07	<0.001	0.06	10.75	0.46
indegree-popularity	0.13	0.01	<0.001	0.12	19.38	0.06
same sex	−0.26	0.11	0.02	0.33	53.82	<0.001
same immigrant origin	0.13	0.07	0.04	0.14	23.08	0.02
Parents’ education similarity	−0.23	0.15	0.12	0.38	25.99	0.01
depressive symptoms alter	−0.04	0.03	0.20	0.00	11.54	0.40
depressive symptoms ego (Hypothesis 3)	0.29	0.10	0.01	0.31	63.78	<0.001
depressive symptoms similarity	−0.02	0.14	0.91	0.30	16.81	0.11

*Note*. Sigma is an estimator for the standard deviation; Q = chi-squared test statistic. Qp < 0.05 indicates that a parameter is significantly different across the population.

## Data Availability

Data are contained within the article and Supplementary Materials.

## Supplementary Materials

The following supporting information can be downloaded at: https://www.mdpi.com/article/10.3390/bs14111110/s1, Table S1: Covariates per class (included and excluded classes); Table S2: Description of liking and disliking networks and depressive symptoms per time point and periods; Table S3: SAOM results for liking and disliking networks for excluded classrooms (N = 10).
