# Supplementary material for: Adolescent Depressive Symptoms and Peer Dynamics: Distorted Perceptions in Liking and Disliking Networks
Source: Behav Sci (Basel). 2024 Nov 19;14(11):1110. doi: 10.3390/bs14111110 (PMC11591119; doi:10.3390/bs14111110)
Supplement: Supplementary file 1 [file behavsci-14-01110-s001.zip › behavsci-3165433-supplementary.pdf]

Table S1. Covariates per class (included and excluded classes)

class	sex mean	age mean	nat mean	edu mean	sex sd	age sd	nat sd	edu sd	dep mean	dep sd
3	1.64	11.52	0.55	3.56	0.49	0.68	0.51	1.38	2.58	0.62
4	1.64	11.73	0.82	3.00	0.50	0.65	0.40	1.55	2.39	0.85
5	1.10	11.70	0.80	3.30	0.32	0.95	0.42	1.42	2.76	0.49
6	1.57	11.43	0.57	4.00	0.53	1.13	0.53	1.67	2.37	0.63
7*	1.37	11.65	0.47	3.87	0.50	0.86	0.51	1.06	2.12	0.66
8	1.43	11.62	0.36	3.46	0.51	0.51	0.50	0.97	2.63	0.54
9*	1.59	11.59	0.29	3.53	0.51	0.51	0.47	1.64	2.17	0.62
10	1.40	11.57	0.33	3.47	0.51	0.65	0.49	1.46	2.62	0.51
11	1.50	11.45	0.42	4.30	0.52	0.93	0.51	0.67	2.53	0.65
12	1.64	11.29	0.29	3.93	0.50	0.47	0.47	0.62	2.43	0.73
13*	1.60	11.60	0.05	4.25	0.50	0.82	0.22	0.85	2.24	0.58
14*	1.52	11.61	0.43	4.13	0.51	0.58	0.51	0.92	2.45	0.61
15	1.50	11.83	1.00	3.88	0.53	0.98	0.00	0.99	2.26	0.97
16	1.50	12.00	0.92	3.50	0.52	1.10	0.29	1.17	2.43	0.97
17	1.50	12.25	0.92	3.82	0.52	1.54	0.29	0.87	2.27	0.64
18*	1.38	11.43	0.33	3.86	0.50	0.81	0.48	1.53	2.64	0.67
19*	1.65	11.45	0.15	4.42	0.49	0.51	0.37	0.96	2.49	0.74
20*	1.48	11.52	0.43	3.75	0.51	0.51	0.51	1.37	2.49	0.57
21*	1.59	11.59	0.65	4.13	0.51	0.62	0.49	1.41	2.67	0.84
22*	1.60	10.83	0.20	4.11	0.50	2.75	0.41	1.29	2.61	0.74
23*	1.57	11.71	0.57	3.53	0.51	0.85	0.51	1.60	2.47	0.65
24*	1.60	11.45	0.65	3.47	0.50	0.60	0.49	1.46	2.21	0.77
25*	1.48	11.52	0.24	4.00	0.51	0.60	0.44	1.21	2.26	0.74
**Av no**	**1.49**	**11.67**	**0.63**	**3.66**	**0.50**	**0.87**	**0.40**	**1.16**	**2.48**	**0.69**
**Av incl**	**1.54**	**11.50**	**0.37**	**3.92**	**0.50**	**0.84**	**0.45**	**1.28**	**2.40**	**0.68**

Notes. * classes included in the analyses; edu: parents' education level; dep: depressive symptoms levels; Av no: Average of no included classrooms; Av incl: Average of included classrooms

Table S2. Description of liking and disliking networks and depressive symptoms per time point and periods

		Liking jaccard		Liking av.degree			Liking ties changes t1-t2			Liking ties changes t2-t3			Disliking jaccard		Disliking av.degree			Disliking ties changes t1-t2			Disliking ties changes t2-t3		
**class**	**n**	**t1-t2**	**t2-t3**	**t1**	**t2**	**t3**	**0-1**	**1-0**	**1-1**	**0-1**	**1-0**	**1-1**	**t1-t2**	**t2-t3**	**t1**	**t2**	**t3**	**0-1**	**1-0**	**1-1**	**0-1**	**1-0**	**1-1**
3	25	.48	.39	11.76	8.96	8.44	126	168	182	88	101	123	0.25	0.23	2.36	3.84	4.08	65	28	31	46	64	32
4	18	.39	.58	5.50	7.94	6.78	33	66	103	25	46	97	0.31	0.41	1.67	2.00	2.61	19	15	15	23	12	24
5	17	.25	.44	4.12	5.53	5.24	39	31	93	33	38	56	0.31	0.40	2.29	2.59	3.18	19	21	18	26	16	28
6	20	.22	.64	2.85	6.60	7.20	23	34	121	36	24	108	0.28	0.55	2.20	4.95	4.75	68	13	31	26	30	69
7*	22	.54	.58	12.00	8.86	11.32	104	160	139	86	32	163	0.26	0.38	1.91	1.82	2.45	23	25	17	29	11	25
8	21	.58	.63	7.14	7.05	9.43	41	109	80	57	18	130	0.24	0.39	2.43	2.19	2.95	27	32	19	29	17	29
9*	19	.40	.53	7.32	6.11	6.21	66	73	109	37	35	81	0.26	0.44	2.21	3.47	4.58	44	20	22	40	19	47
10	22	.41	.33	5.77	5.86	6.14	52	75	106	69	63	66	0.15	0.38	1.82	1.68	2.50	29	18	8	23	14	23
11	17	.39	.50	5.00	5.00	5.29	38	47	72	32	27	58	0.41	0.46	2.59	3.35	3.76	25	16	28	26	19	38
12	21	.61	.68	7.62	10.33	9.71	17	143	90	33	46	171	0.33	0.42	1.52	2.19	1.67	26	13	19	11	22	24
13*	24	.45	.47	9.54	9.12	8.00	90	139	170	61	88	131	0.15	0.21	1.17	3.46	2.79	65	14	14	41	57	26
14*	25	.50	.56	9.96	11.64	10.04	69	180	180	57	97	194	0.28	0.39	4.20	4.00	4.00	55	60	45	44	44	56
15	24	.14	.31	0.96	2.67	3.25	13	10	64	42	31	33	0.13	0.16	0.50	1.58	1.54	26	7	5	25	28	10
16	21	.40	.56	4.90	6.95	7.38	32	71	107	47	38	108	0.07	0.24	0.67	1.33	1.14	23	5	2	13	18	10
17	20	.32	.61	2.25	2.80	3.25	19	24	51	19	10	46	0.15	0.33	1.15	1.20	1.40	18	17	6	15	11	13
18*	24	.51	.50	10.5	9.79	11.50	87	165	157	105	64	171	0.34	0.27	3.38	3.42	2.50	41	40	41	30	52	30
19*	25	.58	.61	11.00	13.56	13.08	50	225	164	75	87	252	0.11	0.37	4.04	4.28	4.64	86	80	21	56	47	60
20*	25	.52	.67	11.76	14.68	13.92	68	226	209	62	81	286	0.13	0.23	1.60	2.44	2.28	47	29	11	35	38	22
21*	20	.54	.53	10.65	10.6	8.8	64	149	127	42	78	134	0.49	0.25	1.95	3.10	2.35	29	6	33	26	38	21
22*	23	.60	.64	16.48	12.87	14.74	126	253	169	92	49	247	0.02	0.13	1.39	0.52	0.87	11	29	1	11	9	3

23*	22	.47	.51	11	10.91	8.64	88	154	174	45	95	145	0.25	0.19	3.23	2.55	3.05	31	45	25	44	37	19
24*	23	.44	.59	9.09	11.70	12.57	62	147	184	82	62	207	0.36	0.49	3.83	4.65	5.74	55	36	52	53	28	79
25*	23	.59	.65	12.74	12.87	12.22	74	219	151	53	68	228	0.31	0.49	2.13	2.26	2.87	28	25	24	27	13	39
**Av/S no**	**20.55**	**.38**	**.52**	**5.26**	**6.34**	**6.56**	**57.82**	**39.36**	**70.73**	**43.73**	**40.18**	**90.55**	**0.24**	**0.36**	**1.75**	**2.45**	**2.69**	**31.36**	**16.82**	**16.55**	**23.91**	**22.82**	**27.27**
**Av/S incl**	**22.92**	**.51**	**.57**	**11.00**	**11.06**	**10.92**	**82.08**	**79.00**	**174.17**	**66.42**	**69.67**	**186.58**	**0.25**	**0.32**	**2.59**	**3.00**	**3.18**	**42.92**	**34.08**	**25.50**	**36.33**	**32.75**	**35.58**

Notes. * Classes included in the analyses; Jaccard index refers to tie stability between observations; DP: depressive symptoms levels; Av/S no: Average or Sum of non included classrooms; Av/S incl: Average or Sum of included classrooms;

Table S3. SAOM results for liking and disliking networks for excluded classrooms (N=10)

Effect	est	se	*p*	sigma	Q	Qp
Liking networks						
Rate period 1	19.475	2.037	<.001	4.053	19.298	<.05
Rate period 2	12.581	0.844	<.001	1.618	17.437	.065
outdegree	-1.173	0.081	<.001	0.239	45.932	<.001
reciprocity	-0.204	0.077	<.01	0.206	28.532	<.01
transitive triplets	0.114	0.008	<.001	0.024	59.39	<.001
indegree-popularity	-0.027	0.013	<.05	0.039	51.637	<.001
same sex	0.347	0.034	<.001	0	8.931	.539
same nationality group	-0.097	0.045	<.05	0.095	17.114	.072
parents education similarity	0.069	0.110	.36	0.291	27.991	<.01
depressive symptoms alter	0.029	0.032	.48	0.065	17.242	.069
depressive symptoms ego (H1)	-0.059	0.022	<.01	0	5.336	.868
depressive symptoms similarity (H2)	19.475	2.037	.03	0.237	18.187	.052
Disliking networks						
Rate period 1	6.726	0.878	<.001	2.183	35.259	<.001
Rate period 2	6.21	0.708	<.001	1.916	42.104	<.001
outdegree	-1.281	0.082	<.001	0.231	44.987	<.001
reciprocity	0.325	0.069	<.001	0.077	9.938	.446
indegree-popularity	0.125	0.011	<.001	0.025	19.439	<.05
same sex	-0.208	0.093	<.05	0.258	34.364	<.001
same nationality group	0.153	0.066	<.01	0.147	18.56	<.05
parents education similarity	-0.243	0.151	.109	0.388	25.533	<.01
depressive symptoms alter	-0.04	0.034	.236	0	6.535	.769
depressive symptoms ego (H3)	0.231	0.099	<.05	0.283	50.634	<.001
depressive symptoms similarity	-0.071	0.15	.636	0.307	16.063	.098

Note. Sigma is an estimator for the standard deviation; Q = chi-squared test statistic. Qp < .05 indicates that a parameter is significantly different across the population.
